# Blood Cells Parameters in Second Trimester of Pregnancy and Gestational Diabetes Mellitus: A Systematic Review and Meta‐Analysis

**DOI:** 10.1002/edm2.70024

**Published:** 2025-01-03

**Authors:** Vida Ghasemi, Mojdeh Banaei, Zahra Kiani, Fahimeh Ramezani Tehrani, Marzieh Saei Ghare Naz

**Affiliations:** ^1^ Department of Public Health Asadabad School of Medical Sciences Asadabad Iran; ^2^ Mother and Child Welfare Research Center Hormozgan University of Medical Sciences Bandar Abbas Iran; ^3^ Midwifery and Reproductive Health Research Center Shahid Beheshti University of Medical Sciences Tehran Iran; ^4^ Reproductive Endocrinology Research Center Research Institute for Endocrine Sciences, Shahid Beheshti University of Medical Sciences Tehran Iran; ^5^ Foundation for Research & Education Excellence Vestavia Hills Alabama USA

**Keywords:** blood parameters, gestational diabetes, haemoglobin, meta‐analysis, platelet, white blood cells

## Abstract

**Objective:**

Gestational diabetes mellitus (GDM) is one of the most common complications during pregnancy. There is inconsistency between previous studies regarding the blood and inflammatory parameters levels among pregnant women and its association with GDM. This study aimed to investigate the relationship between blood parameters in relation to GDM.

**Methods:**

Systematic literature searches were carried out through databases like PubMed, Web of Science, Epistemonikos, Scopus, Scientific Information Database and Magiran till May 2024. The effect size of serum blood parameters levels was determined by using standard mean deviations (SMDs) and 95% confidence intervals (CIs).

**Results:**

Thirty articles were included in this study. Meta‐analysis showed that compared with the control group, women with GDM had significantly higher levels of haemoglobin (0.343 [95% CI 0.134–0.553] *p* = 0.01, sensitivity analysis: 0.174 [95% CI 0.0343–0.315] *p* = 0.01), red blood cell (0.387 [95% CI 0.224–0.550] *p* < 0.001), mean platelet volume (0.498 [95% CI 0.165–0.831] *p* = 0.003), white blood cell count (0.351 [95% CI 0.0882–0.615] *p* = 0.009) and neutrophil–lymphocyte ratio (0.534 [95% CI 0.127–0.941] *p* = 0.01). However, women with GDM had lower levels of mean corpuscular haemoglobin concentration (−0.249 [95% CI −0.386–−0.112] *p* < 0.001). Pooled results from MPV showed no association between adjusted MPV and GDM (adjusted OR 1.33 [95% CI 0.8–1.86] *I* ^2^ = 57.2%).

**Conclusions:**

Finding supports higher levels of blood parameters (Hb, RBC, WBC, NLR and MPV) among women with GDM in the second trimester of pregnancy. Future studies should investigate the potential role of these haematological markers as predictive tools for adverse pregnancy outcomes and evaluate therapeutic interventions targeting these parameters to enhance maternal and fetal health.

## Graphical abstract

Women with GDM in second trimester of pregnancy compared with non‐GDM pregnant women experienced alteration in their blood parameter cells.

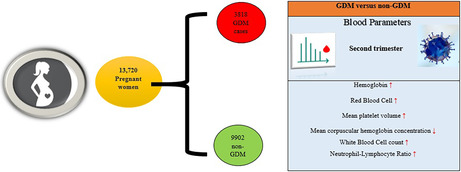

## 1 Introduction

Gestational diabetes mellitus (GDM) is one of the most common complications during pregnancy, which refers to the diagnosis of diabetes in the second and third trimester of pregnancy in the absence of obvious diabetes before pregnancy [1]. According to the latest meta‐analysis study, the prevalence of GDM in Europe was 5.4% [2]. The overall mean prevalence of GDM in the United States and Canada was reported to be 6.9% [3]. GDM is known as a multifactorial disease; genetic, hormonal, inflammatory and immunologic factors contribute to the development of GDM [4]. GDM is also linked to increased risk of adverse maternal and neonatal outcomes [5]. Screening and diagnosis of GDM has been done using one‐step or two‐step approaches [6]. The results of recent meta‐analysis indicated that different diagnostic approaches for GDM could not show difference in incidence rate of the adverse neonatal and maternal outcome [7, 8]. Evidence showed that various biomarkers including inflammatory markers, adipokines, endothelial function and lipids have contributed to the development of GDM [9]. Numerous studies have demonstrated changes in haematological and inflammatory parameters following GDM; however, these studies often present contradictory findings. Liu et al. [10] found that neutrophil‐to‐lymphocyte ratio value (NLR) and the platelet‐to‐lymphocyte ratio (PLR) were higher in the GDM group than in the control group, and Dou et al. [11] reported elevated white blood cell (WBC) counts in the GDM group as well. By contrast, Cirakli et al. [12] did not find any statistically significant differences in NLR, PLR or WBC between the GDM and control groups. Also, Dong et al.'s study showed that platelet parameters like mean platelet volume (MPV) and platelet distribution width (PDW) remained unchanged in mid‐pregnancy in women with GDM compared to those with normal glucose tolerance (NGT) [13]. However, the results of Liu et al. [10] study showed that MPV was significantly higher in pregnant women with GDM than in healthy women. Basu et al. [14] reported that higher levels of haemoglobin and red blood cells (RBCs) were associated with increased risks of GDM, but on the contrary, there was no significant relationship between RBC and haemoglobin with GDM in study conducted by Sahbaz et al. [15].

The conflicting results of studies suggest that further research is needed to clarify the association between haematological and inflammatory parameters and GDM. Considering that the goal of a systematic review and meta‐analysis is to achieve accurate, reliable and high‐quality results from the vast amount of information obtained from primary studies and given the lack of a comprehensive systematic review on this topic, it appears necessary to conduct a thorough systematic review and meta‐analysis to obtain definitive and clear conclusions. So, this study was conducted aimed to assess the blood cell parameters of the second trimester of pregnancy and GDM.

## 2 Methods

This study was conducted according to the meta‐analysis of observational studies in epidemiology (MOOSE) criteria [16]. The search for potential peer‐reviewed publications was carried out through databases like PubMed, Web of Science, Epistemonikos, Scopus, Scientific Information Database (SID) and Magiran till May 2024. Observational studies, including cross‐sectional, case–control and cohort studies with data on confirmed GDM, data on haematological and inflammatory changes in both GDM and healthy controls, in Persian and English without any time restriction were included in this study. Studies were excluded if they were other study designs such as reviews, letter to editor, meeting abstracts, case reports and published in languages other than English and Persian.

The search was conducted using combination of searching terms related to the blood cell parameters and gestational diabetes with OR and AND (Table S1).

In addition, references from pertinent studies were thoroughly examined to identify any additional research that may be relevant to the topic at hand. All output of searches in databases was included in Endnote X7, and after removing duplicates, title‐abstract screening of the remaining records was performed. Then full text of eligible articles was assessed.

### 2.1 Systematic Review Question

In pregnant women (participants) what is the relationship between blood parameters levels (exposures) and GDM (status/outcomes). Inclusion criteria followed PECOT framework including:

- Participant: pregnant women.
- Exposure: blood parameters levels.
- Comparison: healthy pregnant group.
- Outcome: GDM.
- Study type: observational studies.

And at least one of erythrogram, platelets and white blood cell parameters was reported.

### 2.2 Quality Assessments

The Newcastle–Ottawa Scale which covers three dimensions including selection, comparability and exposure/outcome for quality assessment of observational studies [17]. The total score ranged between 0 and 9, the higher the score, the better the quality of included studies. The scores for low, moderate and high‐quality studies were assigned with scores of 0–3, 4–6 and 7–9.

### 2.3 Data Extraction

All information was extracted manually and independently by two researchers. Details of selected studies including participant numbers, participant's baseline age, body mass index (BMI), family history of diabetes, type of study, authors, reference, country, GDM diagnostic criteria and year of publication study design were imported in SPSS version.

### 2.4 Statistical Analyses

The effect size of serum blood parameters levels was determined by using standard mean deviations (SMDs) and 95% confidence intervals (CIs). The mean difference between the case and control groups was taken into account when subtracting the pooled SDs to calculate the SMDs. For studies with reported odds ratios, the pooled results are expressed as OR with corresponding 95% CI. The *I* ^2^ statistics was employed to assess statistical heterogeneity among studies, with a threshold of 25%, 50% and 75% representing low, moderate and high degrees of heterogeneity, respectively [18]. In cases with moderate‐to‐high heterogeneity, the random‐effect model was used to pool all outcomes; otherwise, fixed models were applied for pooled estimates. To investigate the source of heterogeneity, meta‐regression was applied.

The Begg and Egger tests were used to determine if there was potential bias in publishing, with a 0.05 level of significance. All analyses were performed with and STATA statistical software package version 13.0.

## 3 Results

**FIGURE 1:**
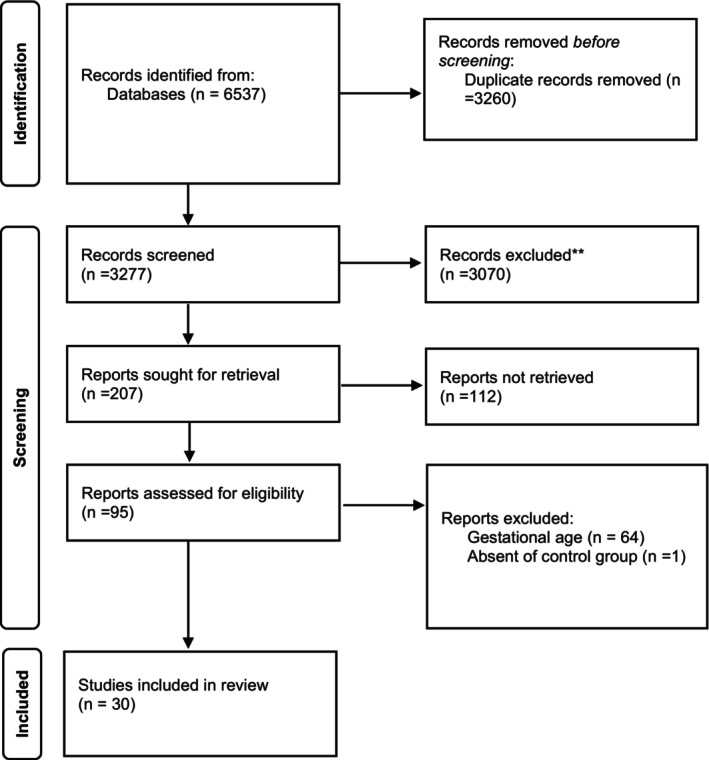
Study selection diagram.

Figure 1 shows the study selection process. An overview of 30 observational studies, with a total of 13,720 individuals (3818 GDM cases and 9902 non‐GDM cases) is provided in Tables 1, 2, 3. The most common diagnostic criteria for GDM in the included studies was IADPSG (25.8%). Fifty studies were conducted in Asia, 12 in Eurasia, 2 in Europe and 1 in Africa. Of all included studies, one study had cross‐sectional design [19], 22 case–control [10, 12, 14, 20, 21, 22, 23, 24, 25, 26, 27, 28, 29, 30, 31, 32, 33, 34, 35, 36, 37, 38], and 7 had cohort design [11, 13, 39, 40, 41, 42, 43]. The age of the participants ranged from 26 to 30 years. The BMI of participants ranged between 21 and 30 kg/m^2^, concerning the family history of diabetes, among included studied 16%–76% of women with GDM and 7%–79% of healthy women reported have family history of diabetes. Summary of results are presented in Table 4.

**TABLE 1:** Haematological factors and related indicators in women GDM and non‐GDM.

Authors/year	Country	Type of study	Sample size	Age (Y) (Mean ± SD)	HCT (Mean ± SD)	MCV (Mean ± SD)	MCH (Mean ± SD)	MCHC (Mean ± SD)	HGB (Mean ± SD)	RBC (Mean ± SD)	RDW (Mean ± SD)	Crude odd	Adjust odd
Zhu et al. 2018^a^	China	Cohort	418	27.90 ± 4.30	—	—	—	—	11.50 ± 0.96	—	—	HB:1.04 (0.99–1.10)	HB:0.96 (0.92–1.02)
			2726	26.20 ± 3.50	—	—	—	—	11.45 ± 0.90	—	—	—	—
Cakmak et al. 2019^b^	Turkey	Case–control	60	29.40 ± 5.42	33.09 ± 4.48	—	—	—	11.04 ± 1.52	—	—	—	—
			75	28.60 ± 5.40	32.56 ± 2.96				10.84 ± 1.16				
Afkhami ardakani and Rashidi, 2009^c^	Iran	Case–control	34	—	—	85.34 ± 10.80	—	—	13.39 ± 1.10	—	—	—	—
			34	—		77.69 ± 6.46			11.75 ± 1.43				
Basu et al. 2018^d^	India	Case–control	114	—	34.34 ± 5.17	87.90 ± 6.90	28.15 ± 3.16	31.80 ± 1.71	11.35 ± 1.22	4.14 ± 0.42	—	—	—
			114	—	32.63 ± 3.22	86.80 ± 7.90	28.09 ± 3.26	32.20 ± 1.58	10.89 ± 1.15	3.87 ± 0.39			
Gorar et al. 2017^b^	Turkey	Cross–sectional	110	—	—	85.10 ± 7.00	—	—	11.80 ± 1.00	—	—	—	—
			159			85.70 ± 7.10			11.60 ± 1.10	—			
Abbasi fashami et al. 2019^e^	Iran	Case–control	95	29.07 ± 6.00	36.60 ± 3.06	86.81 ± 6.17	29.06 ± 2.52	33.42 ± 1.36	12.18 ± 0.93	4.22 ± 0.46	13.90 ± 1.35	—	HCT:1.17 (1.059–1.306)
			95	28.20 ± 6.23	35.34 ± 2.73	88.79 ± 5.20	30.00 ± 2.12	33.80 ± 1.46	11.91 ± 0.82	3.90 ± 0.38	13.30 ± 1.06		MCV:0 0.940 (0.885–0.997)
													RDW:1.450 (1.906–1.112)
ERDOĞAN et al. 2014^f^	Turkey	Case–control	68	31.84 ± 5.67	—	—	—	—	—	—	14.52 ± 1.37	—	—
			61	25.57 ± 5.30							16.12 ± 2.87		
Ahmadi et al. 2021^a^	Iran	Case–control	40	30.70 ± 5.10	—	—	—	—	11.27 ± 0.95	—	—	—	—
			40	27.70 ± 5.80					11.28 ± 0.75				
Cirakli et al. 2022^a^	Turkey	Case–control	214	31.25 ± 7.79	33.75 ± 3.16	82.00 ± 12.10	—	—	11.05 ± 1.94	—	—	—	—
			476	30.75 ± 8.37	34.39 ± 2.81	86.25 ± 12.40			11.60 ± 1.91				
Duo et al. 2024^a^	China	Cohort	258	32.00 ± 1.74	—	—	—	—	11.79 ± 0.90	3.80 ± 0.24	—	—	—
			769	30.25 ± 1.46					11.60 ± 0.35	3.72 ± 0.23			
Adam et al.2017^a^	South Africa	Prospective cohort	143	28.4 ± 8.36					12.5 ± 2.47				
			411	26.8 ± 7.79					12.30 ± 3.19				
Eidgahi et al. 2022^b^	Iran	Cohort	49	28.35 ± 3.89	36.87 ± 2.54	—	—	—	12.32 ± 0.90	4.19 ± 0.41	—	—	—
Bahar Gur et al. 2015^f^	Turkey	Case–control	16	29.00 ± 3.10	—	—	—	—	12.10 ± 0.80	—	—	—	—
Javadian et al. 2014^c^	Iran	Case–control	52	31.24 ± 6.71	37.60 ± 4.20	—	—	—	12.90 ± 0.10	—	—	—	—
Oztekin et al. 2021^c^	Turkey	Case–control	25	29.96 ± 5.48	35.06 ± 2.83	—	—	—	11.66 ± 1.15	—	—	—	—
Şahin Uysal et al. 2020^g^	Turkey	Case–control	273	31.00 ± 6.92	36.25 ± 4.91	—	—	—	12.13 ± 1.26	—	—	—	—
Zhang et al. 2021	China	Longitudinal case control	448	30.60 ± 4.00	—	—	—	—	—	3.82 ± 0.43	—	—	—
KÖs¸Üs et al. 2010^f^	Turkey	Case–control	45	31.20 ± 5.10	34.30 ± 2.30	—	—	—	12.00 ± 1.40	4.25 ± 0.50	13.40 ± 1.00	—	—
Ashraf et al. 2022^c^	Pakistan	Case–control	50	28.90 ± 4.50	—	—	—	—	11.60 ± 2.40	—	—	—	—
Berglund et al. 2016^f^	Spain	Cohort	76	33.70 ± 4.60	—	89.30 ± 4.30	—	—	11.60 ± 0.80	—	—	—	—
Sharifi et al. 2022^e^	Iran	Case–control	64	30.00 ± 4.70	—	—	—	—	12.80 ± 0.80	—	—	—	—
Patxot et al. 2022	Switzerland	Cohort	106	—	34.60 ± 0.75	86.40 ± 1.43	29.00 ± 0.61	33.57 ± 0.36	11.64 ± 0.3	14.00 ± 0.31	—	—	—
			1710		34.30 ± 0.83	87.90 ± 1.42	29.60 ± 0.67	33.66 ± 0.37	11.52 ± 0.43	13.80 ± 0.38	—	—	—

*Note:* In each row, the first row for GDM and the second row non‐GDM.

Abbreviations: HCT, haematocrit; HGB, haemoglobin; MCH, mean corpuscular haemoglobin; MCHC, mean corpuscular haemoglobin concentration; MCV, mean corpuscular volume; RBC, A red blood cell; RDW, red blood cell distribution width.

^a^ IADPSG: The International Association of Diabetes and Pregnancy Study Groups.

^b^ C&C: Carpenter and Coustan.

^c^ ADA: The American Diabetes Association.

^d^ DIPSI: Diabetes in Pregnancy Study Group India.

^e^ WHO: World Health Organization.

^f^ NDDG: National Diabetes Data Group.

^g^ IADPGS: The International Association of Diabetes and Pregnancy Study Groups.

**TABLE 2:** Platelet factors and related indicators in women GDM and non‐GDM.

Authors/year	Country	Type of study	Sample size	Age (Y) (Mean ± SD)	PLT (Mean ± SD)	MPV (Mean ± SD)	PDW (Mean ± SD)	Crude odd	Adjust odd
Liu et al. 2020^a^	China	Case–control	58	29.80 ± 5.10	220.00 ± 32.30	8.50 ± 1.20	—	MPV:3.2 (1.5–4.5)	MPV:4 (1.2–12)
			62	30.20 ± 4.80	205.00 ± 25.80	8.10 ± 0.90			
Basu et al. 2018^b^	India	Case–control	114	—	230.00 ± 73.80	11.90 ± 1.49	17.27 ± 3.05	—	—
			114	—	249.00 ± 77.30	10.42 ± 0.70	16.69 ± 2.78		
Gorar et al. 2017^c^	Turkey	Cross–sectional	110	—	246.70 ± 68.30	—	—	—	PLT: 1.00 (1.000–1.000)
			159	—	227.80 ± 64.20				MPV: 1.005 (0.635–1.539)
ERDOĞAN et al. 2014^d^	Turkey	Case–control	68	31.84 ± 5.67	214.62 ± 67.91	10.50 ± 2.94	16.19 ± 2.42	—	—
			61	25.57 ± 5.30	239.26 ± 90.37	11.15 ± 1.14	14.56 ± 2.80		
Abbasi fashami et al. 2020^e^	Iran	Case–control	110	29.30 ± 5.90	233.00 ± 62.60	10.22 ± 1.50	—	—	PLT:1.01 (1.009–1.022)
			110	27.90 ± 6.20	193.30 ± 49.50	9.45 ± 1.58			MPV: 1.560 (1.267–1.930)
Çeltik et al. 2016^f^	Turkey	Case–control	105	33.41 ± 4.94	253.77 ± 69.43	8.66 ± 1.15	—	—	—
			40	33.32 ± 3.82	253.52 ± 60.43	8.27 ± 0.92			
Cirakli et al. 2022^a^	Turkey	Case–control	214	31.25 ± 7.79	259.25 ± 101.91	9.52 ± 2.97	—	—	—
			476	30.75 ± 8.37	280.75 ± 127.89	9.45 ± 3.14			
Dong et al. 2019^a^	China	Cohort	197	30.45 ± 3.35	245.50 ± 51.24	9.50 ± 1.30	13.60 ± 2.52	—	—
			192	28.70 ± 3.12	237.00 ± 52.59	9.30 ± 1.46	13.90 ± 2.53		
Duo et al. 2024^a^	China	Cohort	258	32.00 ± 1.74	228.78 ± 52.22	—	—	—	—
			769	30.25 ± 1.46	214.82 ± 19.54				
Bahar Gur et al. 2015^d^	Turkey	Case–control	16	29.00 ± 3.10	228.00 ± 34.00	10.90 ± 1.0	—	—	—
			167	28.70 ± 5.00	235.00 ± 52.00	9.60 ± 1.0			
Oztekin et al. 2021^f^	Turkey	Case–control	25	29.96 ± 5.48	252.64 ± 58.73	—	—	—	—
			24	27.75 ± 4.07	266.29 ± 64.32				
Şahin Uysal et al. 2020^g^	Turkey	Case–control	273	31.00 ± 6.92	251.50 ± 96.71	9.65 ± 2.83	—	—	—
			455	28.50 ± 6.93	265.25 ± 122.69	10.27 ± 3.05			
Zhang et al. 2021	China	Longitudinal case control	448	30.60 ± 4.00	237.25 ± 51.55	—	—	—	—
			433	27.00 ± 3.30	219.00 ± 47.69				
Kebapcilar et al. 2016^c^	Turkey	Case–control	101	26.12 ± 5.05	243.80 ± 64.80	9.16 ± 1.04	—	—	—
			138	26.51 ± 5.14	251.60 ± 45.00	7.42 ± 0.80			
KÖs¸Üs et al. 2010^d^	Turkey	Case–control	45	31.20 ± 5.10	236.40 ± 59.30	8.67 ± 1.43	15.90 ± 2.00	—	—
			239	29.60 ± 4.80	253.20 ± 68.00	8.19 ± 0.85	16.10 ± 1.50		
Patxot et al. 2022	Switzerland	Cohort	106	—	226.10 ± 29.01	10.70 ± 0.35	—	—	—
			1710	—	227.40 ± 21.36	10.60 ± 0.38	—	—	—

*Note:* In each row, the first row for GDM and the second row non‐GDM.

Abbreviations: MPV, mean platelet volume; PDW, platelet distribution width; PLT, platelet.

^a^ IADPSG: The International Association of Diabetes and Pregnancy Study Groups.

^b^ DIPSI: Diabetes in Pregnancy Study Group India.

^c^ C&C: Carpenter and Coustan.

^d^ NDDG: National Diabetes Data Group.

^e^ WHO: World Health Organization.

^f^ ADA: The American Diabetes Association.

^g^ IADPGS‐CC: The International Association of Diabetes and Pregnancy Study Groups.

**TABLE 3:** Immunological factors and related indicators in women GDM and non‐GDM.

Authors/year	Country	Type of study	Sample size	Age (Y) (Mean ± SD)	WBC (Mean ± SD)	Lymphocyte (Mean ± SD)	Lymphocyte.P (Mean ± SD)	Neutrophils (Mean ± SD)	Neutrophils.P^g^ (Mean ± SD)	Monocytes (Mean ± SD)	NLR (Mean ± SD)	PLR (Mean ± SD)	Crude odd	Adjust odd
Wang et al. 2020^a^	China	Case–control	147	—	9.52 ± 1.99	1.82 ± 1.30	—	7.21 ± 1.69	—	0.49 ± 0.13	4.36 ± 1.33		NLR: 2.054 (1.66–2.54)	—
			161	—	8.72 ± 1.79	1.81 ± 0.45		6.34 ± 1.48		0.46 ± 1.30	3.62 ± 0.91			
Cakmak et al. 2019^b^	Turkey	Case–control	60	29.40 ± 5.42	—	—	—	—	—	—	3.48 ± 1.31	136.34 ± 43.29	—	—
			75	28.60 ± 5.40							2.94 ± 0.85	112.15 ± 26.04		
Liu et al.2020^c^	China	Case–control	58	29.80 ± 5.10	6.60 ± 1.10	1.70 ± 0.40	—	6.00 ± 1.20	—	—	3.40 ± 0.90	140.30 ± 52.50	WBC: 2.20 (1.30–3.00)	WBC:1.60 (1.20–2.50)
													NLR: 6.5 (3–15)	NLR: 5.5 (1.5–20)
			62	30.20 ± 4.80	6.20 ± 0.80	1.50 ± 0.50		5.40 ± 0.90			3.00 ± 0.80	120.60 ± 40.90	PLR:3.40 (2.10–5.50)	PLR:4.10(1.10–13.10)
Topdagi et al. 2023^c^	Turkey	Case–control	300	35.58 ± 1.56	—	—	—	—	—	—	2.78 ± 1.40	149.65 ± 70.20	—	—
			300	34.41 ± 1.90							1.59 ± 1.20	89.10 ± 31.30		
Basu et al.2018^d^	India	Case–control	114	—	11.95 ± 2.51	—	—	—	—	—	2.87 ± 0.91	—	—	—
			114	—	9.09 ± 1.90						1.79 ± 0.51			
Gorar et al.2017^b^	Turkey	Cross—sectional	110	—	9.80 ± 2.60	—	22.30 ± 7.00	—	68.80 ± 8.30	—	—	—	—	—
			159		9.20 ± 2.80		23.00 ± 7.10		68.30 ± 7.80					
Abbasi Fashami et al. 2020^a^	Iran	Case–control	110	29.30 ± 5.90	9.44 ± 2.00	—	24.80 ± 7.10	—	69.80 ± 7.80	—	3.25 ± 1.96	—	—	WBC:1.13 (0.99–1.37)
									71.10 ± 7.00					
			110	27.90 ± 6.20	8.44 ± 1.90		24.90 ± 7.00				3.06 ± 1.04	—		
Cirakli et al. 2022^c^	Turkey	Case–control	214	31.25 ± 7.79	10.81 ± 2.79	2.18 ± 0.91	—	8.05 ± 2.51	—	—	4.02 ± 1.27	133.25 ± 60.34	—	—
			476	30.75 ± 8.37	10.65 ± 2.89	2.30 ± 0.97		7.91 ± 2.61			4.27 ± 1.78	128.75 ± 54.57		
Duo et al. 2024^c^	China	Cohort	258	32.00 ± 1.74	9.52 ± 0.86	—	—	—	—	—	—	—	—	—
			769	30.25 ± 1.46	9.52 ± 0.80									
Oztekin et al. 2021^e^	Turkey	Case–control	25	29.96 ± 5.48	12.16 ± 1.77	—	—	—	—	—	—	—	—	—
			24	27.75 ± 4.07	10.54 ± 1.72									
Şahin Uysal et al.2020^f^	Turkey	Case–control	273	31.00 ± 6.92	10.59 ± 4.34	2.15 ± 1.17	—	7.77 ± 3.53	—	0.67 ± 0.48	5.85 ± 3.77	—	—	—
			455	28.50 ± 6.93	12.77 ± 6.06	2.49 ± 1.46		10.11 ± 5.59		0.67 ± 0.43	7.11 ± 5.20			
Zhang et al. 2021	Turkey	Longitudinal case control	448	30.60 ± 4.00	10.34 ± 2.28	—	—	7.55 ± 1.94	—	—	—	—	—	—
			433	27.00 ± 3.30	9.51 ± 2.30			6.91 ± 2.02						
Yilmaz et al.2014^b^	Turkey	Case–control	42	30.42 ± 3.41	—	—	—	—	—	—	3.00 ± 0.83	—	NLR: 6.982 (3.04–16.05)	NLR: 5.51 (1.35–22.48)
			68	26.75 ± 3.19	—						2.26 ± 0.43			
Patxot et al. 2022	Switzerland	Cohort	106	—	—	1.90 ± 0.33	—	7.30 ± 0.89		0.70 ± 0.22				
			1710	—	—	1.80 ± 0.45	—	6.90 ± 0.86		0.70 ± 0.22				

*Note:* In each row, the first row for GDM and the second row non‐GDM.

Abbreviations: NLR, neutrophil‐to‐lymphocyte ratio; PLR, platelet‐to‐lymphocyte ratio; WBC, white blood cells.

^a^ WHO: World Health Organization.

^b^ C&C: Carpenter and Coustan.

^c^ IADPSG: The International Association of Diabetes and Pregnancy Study Groups.

^d^ DIPSI: Diabetes in Pregnancy Study Group India.

^e^ ADA: The American Diabetes Association.

^f^ IADPGS‐CC: The International Association of Diabetes and Pregnancy Study Groups.

g Neutrophils (%).

According to the NOS, all of the included studies had moderate‐to‐high quality. The mean (SD) of quality score of included studies was 7.97 (1.01) (Figure S1).

### 3.1 Erythrogram and GDM

**FIGURE 2:**
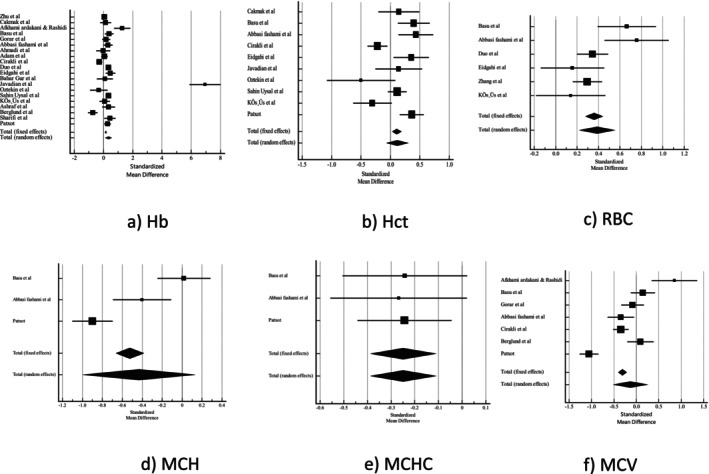
Forest plot of (a) Hb, (b) Hct, (c) RBC, (d) MCH, (e) MCHC, (f) MCV. Abbreviations: Hb, haemoglobin concentration; Hct, haematocrit; MCV, mean corpuscular volume; MCHC, mean corpuscular haemoglobin concentration; MCH, mean corpuscular haemoglobin; RBC, red blood cell.

**FIGURE 3:**
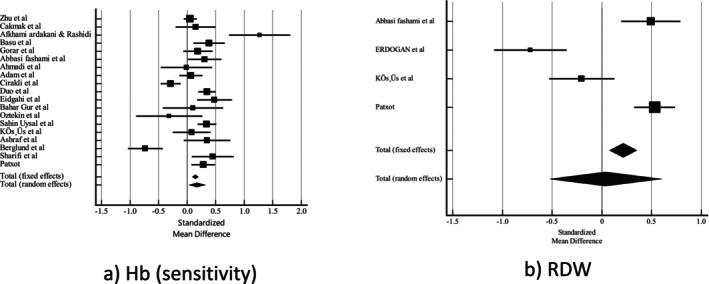
Forest plot of (a) Hb (sensitivity), (b) RDW.

Employing a random‐effects model, we assessed the standardised mean differences with 95% CI for Hb, Hct, MCV, MCH, MCHC, RBC and RDW (Table 4). Pooled analysis showed that women with GDM had significantly higher Hb levels (0.343 [95% CI 0.134–0.553] *p* = 0.01, sensitivity analysis: 0.174 [95% CI 0.0343–0.315] *p* = 0.01) and RBC (0.387 [95% CI 0.224–0.550] *p* < 0.001) compared with the control group. However, women with GDM had lower levels of MCHC (−0.249 [95% CI −0.386–−0.112] *p* < 0.001) than the control group. There were no significant differences for Hct, MCV, MCH and RDW (Figures 2 and 3).

**TABLE 4:** Pooled standard mean differences of blood cell parameters, heterogeneity and publication bias results.

Variable	Study	N1	N2	Total	SMD	95% CI	*p*	Test for heterogeneity		Publication bias	
								*I* ^2^ (inconsistency)	*p*	Egger's test	Begg's test
Erythrogram											
Hb	20	2242	8337	10,579	0.343	0.134–0.553	**0.001**	93.34%	*p* < 0.0001	*p* = 0.1070	*p* = 0.6970
Hb sensitivity	19	2190	8287	10,477	0.174	0.0343–0.315	**0.015**	84.68%	*p* < 0.0001	*p* = 0.5995	*p* = 0.8065
HCT	10	1033	3789	4822	0.117	−0.0655–0.300	**0.209**	80.26%	*p* = 0.2547	*p* = 0.8838	*p* = 0.4208
MCV	7	749	2716	3465	−0.132	−0.510–0.246	0.494	93.91%	*p* < 0.0001	*p* = 0.1279	*p* = 0.0985
MCH	3	315	1919	2234	−0.432	−0.995–0.130	0.132	93.63%	*p* < 0.0001	*p* = 0.4165	*p* = 0.6015
MCHC	3	315	1919	2234	−0.249	−0.386–−0.112	**< 0.001**	0%	*p* = 0.9884	*p* = 0.5246	*p* = 0.1172
RBC	6	1009	2201	3210	0.387	0.224–0.550	**< 0.001**	70.02%	*p* = 0.0052	*p* = 0.5841	*p* = 0.8510
PLT measurements											
PLT	16	2248	5149	7397	0.0653	−0.0905–0.221	0.411	86.33%	*p* < 0.0001	*p* = 0.3412	*p* = 0.8571
PDW	4	424	606	1030	0.128	−0.182–0.438	**0.418**	80.38%	*p* = 0.0016	*p* = 0.3335	*p* = 0.1742
RDW	4	314	2105	2419	0.0372	−0.518–0.592	0.895	93.59%	*p* < 0.0001	*p* = 0.1591	*p* = 0.0415
MPV	12	1407	3764	5171	0.498	0.165–0.831	**0.003**	95.33%	*p* < 0.0001	*p* = 0.0408	*p* = 0.0998
Inflammatory parameters											
WBC	11	1757	2763	4520	0.351	0.0882–0.615	**0.009**	93.91%	*p* < 0.0001	*p* = 0.0944	*p* = 0.1797
LYM	5	798	2864	3662	0.0293	−0.184–0.243	0.788	82.23%	*p* < 0.0001	*p* = 0.0909	*p* = 0.1416
MONO	3	526	2326	2852	0.00703	−0.0981–0.112	0.896	0%	*p* = 0.9703	*p* = 0.4461	P = 0.1172
NEUT	6	1246	3297	4543	0.236	−0.0986–0.570	**0.167**	94.97%	*p* < 0.0001	*p* = 0.4157	*p* = 0.3476
NLR	9	1318	1821	3139	0.534	0.127–0.941	**0.010**	96.48%	*p* < 0.0001	*p* = 0.1489	*p* = 0.2109
PLR	4	632	913	1545	0.576	−0.00192–1.154	0.051	96.00%	*p* < 0.0001	*p* = 0.9420	*p* = 1.0000

*Note:* Bold values shows significancy.

Abbreviations: Hb, haemoglobin concentration; Hct, haematocrit; LYM, lymphocyte; MCH, mean corpuscular haemoglobin; MCHC, mean corpuscular haemoglobin concentration; MCV, mean corpuscular volume; MCV, mean corpuscular volume; Mono, monocytes; MPV, mean platelet volume; MPV, mean platelet volume; NEUT, neutrophils; NLR, neutrophil–lymphocyte ratio; PLT, platelet count; RBC, red blood cell; RDW, red cell distribution width; WBC, white blood cells. SMD, standardized mean diffrence; CI 95%, Confidence interval.

### 3.2 PLT Measurements and GDM

**FIGURE 4:**
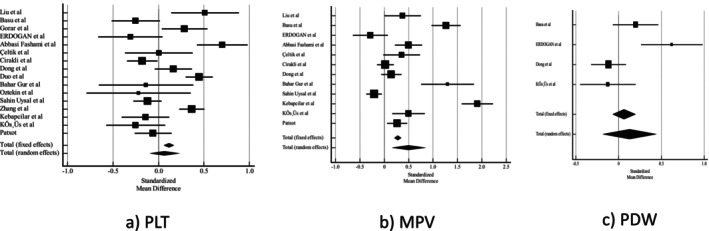
Forest plot of (a) PLT, (b) MPV, (c) PDW. Abbreviations: MPV, mean platelet volume; PDW, platelet distribution width; PLT, platelet count.

As results showed women with GDM had higher levels of MPV than the control group (0.498 [95% CI 0.165–0.831] *p* = 0.003). The results revealed that there was no significant difference in the PLT counts and PDW between two groups. Pooled results from MPV showed no association between adjusted MPV and GDM (adjusted OR 1.33 [95% CI 0.8–1.86, *I* ^2^ = 57.2%]) (Figure 4).

### 3.3 Inflammatory Parameters and GDM

**FIGURE 5:**
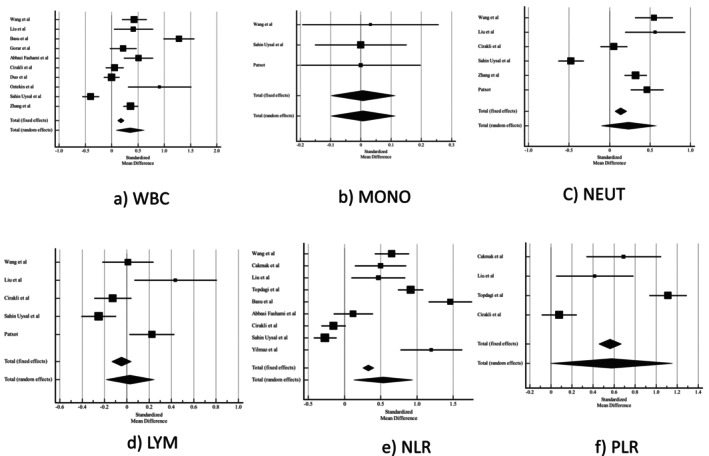
Forest plot of (a) WBC, (b) MONO, (c) NEUT, (d) LYM, (e) NLR, (f) PLR. Abbreviations: LYM, lymphocyte ratio; MONO, monocytes; NEUT, neutrophil; NLR, neutrophil‐to‐lymphocyte ratio; PLR, the platelet–lymphocyte ratio; WBC, white blood cells.

Compared with the control group, women with GDM had higher WBC count (0.351 [95% CI 0.0882–0.615] *p* = 0.009) and NLR (0.534 [95% CI 0.127–0.941] *p* = 0.01). The meta‐analyses revealed that there was no significant difference in the lymphocyte (LYM), monocyte (MONO) and neutrophil (NEUT) counts, and PLR between the two groups. Pooled result of crude NLR showed no association between NLR and GDM (crude OR 4.03 [95% CI 0.43–7.63] *I* ^2^ = 532%) (Figure 5).

### 3.4 Meta‐Regression

Meta‐regression analysis to explore the association between BMI, age, continent and GDM was planned for all outcomes. However, due to insufficient observations for some outcomes, meta‐regression could not be performed for these cases. According to the meta‐regression analysis's findings, BMI differences (*B* = 0.269, *p* = 0.040), age differences (*B* = 0.652, *p* = 0.015) and continent (*B* = 1.545, *p* = 0.004) may be sources of heterogeneity for MPV. However, these variables were not a source of heterogeneity for other parameters including HB, HCT, PLT and NLR.

## 4 Discussion

In the present systematic review and meta‐analysis, we explore the association of second‐trimester blood parameters with GDM. The total number of participants included in this study consists of 3818 GDM cases and 9902 non‐GDM cases. The results showed that GDM is associated with increased levels of Hb, RBC, MPV, WBC and NLR and decreased MCHC. In order to find source of heterogeneity, we did meta‐regression. However, the substantial proportion of heterogeneity remained unexplained. This suggest that other unmeasured factors may know as source of heterogeneity.

GDM is a common metabolic disorder that potentially affects maternal and fetal health [44]. It is proposed that alterations in haematological parameters might be linked to GDM. Recognition the most associated factors of GDM can help to early detection and tailored management of GDM and ultimately improving both short‐ and long‐term outcome [45].

In this study, results demonstrate that, in pregnant women with GDM, the levels of both Hb and RBC are significantly higher than the controls. This underlines an important variation in the haematological profile of patients with GDM, which is clinically relevant. Fernández‐Cao et al. [46] also found the association between high Hb and risk of GDM. Although some of the initial reports regarding levels of Hb and RBC in GDM subjects had presented conflicting results [12, 15, 26, 30, 42], this meta‐analysis that synthesises data from several primary studies forms robust evidence for the increased Hb and RBC levels in women with GDM. Further, results showed that the MCHC was significantly lower in the women with GDM when compared to the ones without GDM. A study among patients with diabetes showed that mean MCHC in patients with Type 2 diabetes was lower than controls [47].

Although the mechanisms by which Hb, RBC, MCHC influence GDM or vice versa are not well understood, researchers suggested some mechanisms for such association; it has been reported that insulin resistance, the hallmark of GDM, may disrupt iron metabolism and erythropoiesis at multiple steps, leading to a suboptimal haemoglobin concentration within the RBCs [48, 49]. On the other hand, GDM is characterised by impaired in glucose metabolism, which increases oxidative stress and subsequent inflammation [50]. Higher oxidative stress may lead to increased oxidation of haemoglobin and also membranes of RBCs, which in turn may reduce the cell capability to maintain a typical haemoglobin concentration [51]. Besides, inflammation could interfere with the process of erythropoiesis, resulting in the production of RBCs of altered haemoglobin content [52]. Generally, alteration in the RBC haemoglobin concentration may insinuate that both oxidative stress and inflammation differentially affect RBC function [46, 50]. Anyway, more studies should be designed to explain how MCHC decreases in this pathology and further study to identify the significance of this phenomenon for GDM treatment. In‐depth exploration of these pathways may assist in improving management strategies for anaemia and other haematological disorders in GDM pregnancies. Our study also found that women with GDM had higher levels of MPV than the control group during the second trimester, similar to the findings of Zhou et al. [53], although their study observed this difference in the third trimester. However, there was no significant difference between the PLT counts in the two groups, suggesting that GDM mainly affects platelet function and activity rather than production [54]. GDM is associated with chronic low‐grade inflammatory disorders [55]. Activated platelets release proinflammatory cytokines and chemokines, establishing a close relationship between inflammation and platelet activation [56]. Among women with GDM, high levels of MPV are known an inflammatory response [57].

Furthermore, our findings showed that WBC count and NLR were significantly higher in women with GDM when compared to the control group, whereas no significant difference existed in NEUT and LYM counts between the two groups. The significant increase in WBC count and NLR among GDM patients reflects the probably associated inflammatory state of GDM [55]. Consistent with our finding, in a meta‐analysis, Pace and Vassallo demonstrated that NLR was higher in women with GDM than in controls [58]. The role of NLR as a marker of systemic inflammation in chronic diseases is well documented, and the detection of its raised levels in GDM points out the importance of monitoring markers of inflammation as a part of the standard management in these patients [59]. This may suggest a selective activation of inflammatory pathways in GDM, further helping refine risk assessments and also guiding inflammation‐targeted interventions. The absence of a difference in NEUT, LYM and MONO counts could be an indication that a generalised inflammatory response does not take place but rather one limited to only some types of WBCs. Further studies may elaborate on the specific mechanisms of inflammation underlying these haematological changes, which can provide newer insights into improving targeted therapies in inflammation‐related complications of GDM.

### 4.1 Strength and Limitation

This study findings have to be interpreted in the light of limitations and strengths. Evaluating the association of multiple blood cell parameters with GDM provides holistic understanding of the relationship of blood cells parameters and GDM. However, there are also some limitations in this study are as follows: heterogeneity among included studies regarding diagnostic criteria for GDM, study design and populations studied, which makes it difficult to draw consistent conclusions and raises concerns about the generalisability of results. The varying diagnostic criteria for GDM create limitations such as inconsistent prevalence estimates, variation in screening approaches, differing blood glucose thresholds and difficulty in comparing research studies. Future research should aim to improve reporting of related covariates in primary studies to enable more comprehensive meta‐regression analyses. Additionally, some studies had small sample sizes which reduced their statistical power. Therefore, it is suggested to conduct longitudinal studies with a large sample size to investigate these haematological and inflammatory changes as well as their predictive role in the development of GDM. This point should also be mentioned, a large number of studies did not report the standard cut‐off of haematological and inflammatory markers and receiver operating characteristic (ROC) results. Future studies also should target the utility value of these markers as predictors of adverse pregnancy outcome and their role as therapeutic guides to improve maternal and fetal outcomes in GDM pregnancies.

## 5 Conclusion

Our findings evidence that GDM is associated with significant changes in haematological parameters like higher levels of Hb, RBC, MPV, WBC and NLR and lower MCHC levels. The recognition of such alteration may be useful for the better management of GDM with minimal risk. The results of our studies may provide valuable insights into the pathophysiology of GDM and by emphasising the predictive role of haematological and inflammatory factors it can improve clinical management strategies, early diagnosis and treatment of GDM. There is a need for further research to establish these haematological markers as predictive indices for the adverse pregnancy outcome and the therapeutic strategy aimed at improving these parameters may help improve maternal and fetal outcome in GDM.

## Author Contributions

Conception or design, acquisition, analysis or interpretation of data: M.S.G.N., V.G. drafting the work or revising, final approval of the manuscript: F.R.T., M.S.G.N., V.G., M.B., Z.K.

## Ethics Statement

The study was approved by the ethical review board of the Research Institute for Endocrine Sciences, Shahid Beheshti University of Medical Sciences, Tehran (IR.SBMU.ENDOCRINE.REC.1403.069).

## Consent

The authors have nothing to report.

## Conflicts of Interest

The authors declare no conflicts of interest.

## Supporting information


Data S1.

## Data Availability

The data sets used and/or analysed during the current study available from the corresponding author upon reasonable request.
