# Supplementary material for: Blood Cells Parameters in Second Trimester of Pregnancy and Gestational Diabetes Mellitus: A Systematic Review and Meta‐Analysis
Source: Endocrinol Diabetes Metab. 2025 Jan 3;8(1):e70024. doi: 10.1002/edm2.70024 (PMC11702436; doi:10.1002/edm2.70024)
Supplement: Supplementary file 1 — Data S1. [file EDM2-8-e70024-s001.docx]

| set | Search strategy PubMed |
| --- | --- |
| #1 | **(Diabetes, Pregnancy-Induced[Title/Abstract]) OR (Diabetes, Pregnancy Induced[Title/Abstract])) OR (Pregnancy-Induced Diabetes[Title/Abstract])) OR (Gestational Diabetes[Title/Abstract])) OR (Diabetes Mellitus,Gestational[Title/Abstract])) OR (Gestational Diabetes Mellitus[Title/Abstract])) OR (GDM[Title/Abstract])** |
| #2 | **(Blood Cell Counts) OR (Count, Blood Cell)) OR (Blood Cell Number)) OR (Blood Cell Numbers)) OR (Blood Count, Complete)) OR (Count, Erythrocyte)) OR (Erythrocyte Counts)) OR (Red Blood Cell Count)) OR (Erythrocyte Number)) OR (Erythrocyte Numbers)) OR (Reticulocyte Counts)) OR (Reticulocyte Number)) OR (Number, Reticulocyte)) OR (Leukocyte Counts)) OR (Leukocyte Number)) OR (Leukocyte Numbers)) OR (White Blood Cell Count)) OR (Differential Leukocyte Count)) OR (Differential Leukocyte Counts)) OR (Lymphocyte Counts)) OR (Lymphocyte Number)) OR (Lymphocyte Numbers)) OR (Total Lymphocyte Count)) OR (Total Lymphocyte Counts)) OR (Platelet Counts)) OR (Platelet Count)) OR (Platelet Number)) OR (Platelet Numbers)) OR (Blood Platelet Number)) OR (Blood Platelet Count)) OR (Blood Platelet Counts)) OR (Neutrophil)) OR (Neutrophils)) OR (Polymorphonuclear Leukocyte)) OR (Polymorphonuclear Leukocytes)) OR (Polymorphonuclear Neutrophils)) OR (Polymorphonuclear Neutrophil)) OR (Neutrophil Band Cell)) OR (Neutrophil Band Cells)) OR (Monocytes)) OR (Lymphocyte)) OR (Lymphoid Cells)) OR (Lymphoid Cell)) OR (Eosinophils)) OR (Basophils)) OR (Hematocrits)) OR (Packed Red-Cell Volume)) OR (Packed Red-Cell Volumes)) OR (Packed Erythrocyte Volume)) OR (Packed Erythrocyte Volumes)) OR (Hemoglobin)) OR (Eryhem)) OR (Ferrous Hemoglobin)** **OR (Mean Cell Volume) OR (Mean Cell Hemoglobin)** **OR (Mean Corpuscular (Cell) Hemoglobin Concentration) OR (Red Cell Distribution Width)** **OR (Nucleated Red Blood Cell)** **OR (Mean Platelet Volume) OR (Platelet Distribution Width)** **OR (Platelet-Large Cell Ratio)** **OR (Platelet Mass Index)** **OR (Plateletcrit)** **OR (Erythrocyte Sedimentation Rate)** **OR (Neutrophil-To-Lymphocyte Ratios)** **OR (Platelet-To-Lymphocyte Ratios))** |
| #3 | #1 AND #2 |

Supplementary Table 1: Search strategy

Supplementary figure 1. Quality assessment of included studies
